# Assessment measures for chemotherapy-induced peripheral neuropathy among pediatric oncology patients: an updated systematic review

**DOI:** 10.1007/s00520-025-09515-5

**Published:** 2025-05-29

**Authors:** Ting Mao, Janelle Yorke, Yan Shi, Nanping Shen, Haixia Wang, Frances-Kam-Yuet Wong, Katherine Ka Wai Lam, Lai Ngo Tang, Qi Liu, Hammoda Abu-Odah, Getaneh Mulualem Belay, Funa Yang, Li Wang, Frankie Wai Tsoi Cheng, Xiaoju Zhang, Ka Yan Ho

**Affiliations:** 1 School of Nursing, The Hong Kong Polytechnic University, Hung Hom, Hong Kong SAR , China; 2 School of Medicine, Tongji University, Shanghai, China; 3 Department of Nursing, Shanghai Children’s Medical Center, Shanghai Jiao Tong University School of Medicine, Shanghai, China; 4 Haematology and Oncology Centre, Hong Kong Children’s Hospital, Kowloon, Hong Kong SAR, China; 5 Shenzhen Children’s Hospital, Shenzhen, China; 6 Department of Nursing, Fudan University Shanghai Cancer Center, Shanghai, China; 7 JC STEM Lab of Digital Oncology Care Enhancement (DOCE), The Hong Kong Jockey Club Charities Trust, Hong Kong SAR, China

**Keywords:** Chemotherapy-induced peripheral neuropathy, Assessment measure, Pediatric oncology, Systematic review

## Abstract

**Objective:**

To update the systematic review of assessment tools on chemotherapy-induced peripheral neuropathy (CIPN) for pediatric oncology patients based on the evidence available after the published review in 2020.

**Data sources:**

Seven English-language databases (PubMed, CINAHL, PsycINFO, EMBASE, the Cochrane Central Register of Controlled Trials, Scopus and Web of Science) were searched for studies published from Nov 9, 2018, to May 20, 2024.

**Study selection:**

Studies that contained subjects who had a cancer diagnosis and were aged under 18 years and discussed the development of a tool to measure CIPN or assessed all test items and response categories for CIPN were included.

**Data extraction and synthesis:**

Data were screened and extracted independently using predesigned tables. The quality of each study was assessed based on Joanna Briggs Institute’s critical appraisal tools for analytical cross-sectional studies and case control studies. The quality of identified instruments for CIPN was evaluated by the modified version of Quality Assessment of Diagnostic Accuracy Studies (QUADAS) tool.

**Results:**

A total of 5 studies (with 633 patients) were included in the systematic review. Only one study was rated as high quality. We newly identified two patient-reported outcome measures (PROMs), one objective assessment and a pain scale, and three new studies about the previous identified CIPN assessment measures.

**Conclusions and relevance:**

Based on the current evidence, the pediatric-modified Total Neuropathy Score (ped-mTNS) and the Total Neuropathy Score-Pediatric Vincristine (TNS-PV) are still the two most appropriate tools for healthcare professionals to use in clinical settings. Our results also addressed the gap in existing literature by showing two newly PROMs for CIPN in pediatric oncology patients with acceptable quality. The combination of physician-based assessment tools and PROMs are recommended to evaluate the patients’ CIPN-related symptoms.

**Supplementary Information:**

The online version contains supplementary material available at 10.1007/s00520-025-09515-5.

## Introduction

Chemotherapy is one of the major treatment modalities for cancer [1]. This treatment involves the use of different chemotherapeutic agents to destroy cancer cells and suppress the tumor growth [2]. However, during chemotherapy, it is inevitable that normal cells are also killed, resulting in various side effects [2]. One of the common side effects raised by cancer patients is chemotherapy-induced peripheral neuropathy (CIPN) which refers to the injury, inflammation, and/or degeneration of peripheral nerve caused by the chemotherapeutic agents [3]. CIPN is manifested in a combination of sensory, motor, and autonomic symptoms in different intensity and duration [4, 5] and often starts in extremities, including fingers and toes and subsequently spread in a glove and stocking distributions [4].

The prevalence of CIPN in adult cancer patients is high, affecting around 19% to over 85% of the population [6]. Some chemotherapeutic agents are known to be having a high risk to cause CIPN, and these agents include platinum-based compounds, taxanes, vinca alkaloids, and thalidomide [7]. Other risk factors of CIPN in adult cancer patients are older age, history of neuropathy, alcohol intake, lower hemoglobin level, and higher body mass index [8, 9]. CIPN is also a common problem in pediatric oncology patients, with the prevalence ranging from 50 to 90%, depending on the chemotherapeutic agents used, accumulative dose, and different risk factors [10]. Compelling evidence shows that pediatric oncology patients with CIPN demonstrate impaired motor functions, including weakness, fasciculation, and muscle atrophy [11], and sensory deficits, such as numbness, tingling, and altered perception of pain [10, 11]. The compromised motor and sensory functions severely intervene the daily activities of pediatric oncology patients, leading to different psychological symptoms and subsequently affecting their quality of life [12].

Despite the severity and seriousness of CIPN in pediatric oncology patients, this problem is usually overlooked and undiagnosed in clinical settings [13]. A major reason is that there is no agreement on the diagnostic tool for CIPN in pediatric oncology patients [14]. The assessment of CIPN in pediatric oncology settings is heavily relied on clinical physicians [15]. Currently, the Total Neuropathy Score (TNS) variants have been commonly used as an objective measure to assess CIPN among children with cancer [15]. Although the pediatric TNS variants have demonstrated strong psychometric properties [14, 16–18], these measures are not feasible for routine use in clinical settings. Factors that limit the use of the pediatric TNS variants include (1) the need for TNS-trained assessors, (2) the time and clinical space for assessments, (3) discomforts associated with some testing procedures, e.g., pin prick, and (4) difficulties of children to focus and cooperate in a lengthy TNS assessment. To overcome these implementation barriers, patient-reported outcome measures (PROMs) which emphasize to collect health outcomes directly from patients without any interpretation from clinicians and other healthcare professionals [19] may be a feasible option to measure CIPN among pediatric oncology patients.

A review of literature revealed two systematic reviews that summarized the assessment measures of CIPN for pediatric patients [20, 21]. However, the 1 st systematic review [20] only included pediatric patients receiving vincristine which limits the exploration of other chemotherapy drugs that cause CIPN. The 2nd systematic review [21] which did not limit the chemotherapy agents was searched on 2018 and published in 2020 and included seven articles published between 2009 and 2018. Their results identified 12 measures, including (1) two pediatric variants of the TNS: Pediatric-Modified Total Neuropathy Score (ped-mTNS) [14, 16, 17, 22] and the Total Neuropathy Score-Pediatric Vincristine (TNS-PV) [18], (2) two grading scales, named the National Cancer Institute Common Terminology Criteria for Adverse Events (NCI-CTCAE)[16, 18] and the Balis Pediatric Scale of Peripheral Neuropathy [18], (3) two objective tests named nerve conduction velocity (NCV) test [23] and quantitative sensory testing including vibration perception threshold (VPT) and tactile perception threshold (TPT) tests, current perception threshold (CPT) test [23], (4) the Wong-Baker FACES pain scale [18], (5) mobility measures, including physical exam, deep tendon reflexes, cranial nerve exam, muscle strength, muscle tone, sensation, and coordination [24], and (6) one balance subscale derived from motor skills scale, named Bruininks-Oseretsky Test of Motor Proficiency version 2 (BOT-2)—Balance Subscale [14, 22].

This systematic review concluded that ped-mTNS and TNS-PV are promising but require further testing [21]. Although this systematic review recommended 12 tools that are valid and reliable to assess CIPN for children with cancer, these tools are not PROMs and are relied on physicians to perform the assessment. Hence, these tools are unable to assess and capture the experience of pediatric oncology patients regarding CIPN notwithstanding the increasing emphasis on patient-centered medicine [25] which integrate PROMs to guide clinical decision making [26]. In fact, a lack of measures to assess pediatric oncology patients and their experience of CIPN is regarded as a major reason to the underestimation of the severity and prevalence of CIPN in this population group[13, 15].

## Descriptions of the identified tools for CIPN

### Pediatric-modified total neuropathy score (Ped-mTNS)

This measure assesses eight signs and symptoms of CIPN using scripted interviews [17]. The sign and symptoms can be grouped into three different categories, including sensory, i.e., numbness, tingling, and pain, motor, i.e., difficulty in buttoning, zipping, walking, and managing stairs, and automatic, i.e., dizziness and hot or cold hands or feet [17]. The trained clinicians assess pediatric oncology patients on these eight signs and symptoms on a 5-point rating scale via light touch, pain sensation, vibration perception, strength, and deep tendon reflexes. All the item scores are summed to give a total score ranging from 0 to 32, with higher scores indicating more severe symptoms and more proximal extension of neurological deficits [18]. The point equal to or more than five indicates the presence of CIPN [14]. In the previous systematic review, five studies provided low to moderate evidence supporting the psychometric properties of the ped-mTNS. Although there is insufficient evidence to support the use of the ped-mTNS to assess CIPN in children, it is still a promising tool for this population group[21].

### Total neuropathy score-pediatric vincristine (TNS-PV)

This scale was developed for adults and then revised for children to capture their vincristine-induced peripheral neuropathy, rather than CIPN induced by other chemotherapeutic agents [18]. The signs and symptoms captured by the TNS-PV are numbness, tingling and neuropathic pain, proximal extension, vibration and warmth sensation, muscle strength, deep tendon reflexes, constipation, and hoarseness/vocal cord function [18]. The TNS-PV contains 7 items rated on 0–4 scales [18] by trained medical personnel (e.g., nurses and physiotherapists) within 5 to 10 min [27]. The original scoring system for TNS-PV is named as Form A in which signs and symptoms experienced in the hands versus the feet are not differentiated [18]. In particular, a patient who only experience CIPN symptoms in the hands receives the same score as the patient who experience CIPN symptoms in both the hands and feet. Hence, another scoring system (Form B) was developed to reflect that CIPN symptoms in both the hands and feet are more severe than the symptoms presented in the hands alone [18]. One study in previous systematic review assessed reliability and validity of TNS-PV and was deemed high quality [27]. But stronger psychometric evidence about the TNS-PV from case–control studies and more diverse children oncology patients is needed.

### National cancer institute common terminology criteria for adverse events (NCI-CTCAE v3.0/v4.0)

The previous systematic indicated that the Common Terminology Criteria for Adverse Events (NCI-CTCAE v3.0[28]/v4.0[29]) was used to assess the pediatric peripheral neuropathy. The CTCAE v3.0/v4.0 quantifies both sensory and motor neuropathy on a 1–5 scale, with 1 = mild adverse event/asymptomatic, 2 = moderate adverse event, 3 = severe adverse event, 4 = life-threatening adverse event, and 5 = fatal adverse event [28, 29]. Study findings about NCI-CTCAE were mixed. No correlation and moderate correlation between combined motor and sensory CTCAE scores and ped-mTNS [16] and TNS-PV [18] were found, respectively. The authors suggested that the sensitivity of the CTCAE to detect subtle CIPN is inferior to that of the ped-mTNS[21].

### Balis pediatric scale of peripheral neuropathy

The Balis Pediatric Scale of Peripheral Neuropathy is similar to CTCAE and requires a physician to assess neuropathy in children on a 4-point rating scale [18]. The Balis scale has a moderate construct validity when compared with the TNS-PV [18]. The previous systematic review concluded that the Balis scale might be useful to assess the general CIPN initially in the clinical settings [21]. However, more sensitive measures are needed to be used to assess CIPN symptoms and severity.

### Nerve conduction velocity (NCV) test

This test is to measure how fast electrical signals can transfer via a motor nerve or a sensory nerve. Through the test, we can assess nerve function and nerve damage [30]. To perform the test, we have to tap electrodes in specific places along a neural pathway [31]. A technician then stimulates the nerve via a mild electrical impulse and records how fast the electrical impulse travels along the neural pathway via the electrodes [31]. The velocity less than 42 m/s in plantar nerve or less than 40 m/s in post tibial nerve in adults is considered as neuropathy [32]. For children, the conduction velocities are half of the values in adults [32]. The previous review did not report the psychometric properties of NCV [21].

### Vibration perception threshold (VPT) and tactile perception threshold (TPT) tests

VPT test is a simple physical examination to examine large sensory nerve fibers by a vibratory sensory analyzer, a quantitative sensory testing computerized device [33]. The VPT is determined by increasing the intensity of a vibration stimulus until the patient can detect the stimulus [34]. Similar to VPT, TPT is to determine the threshold when a patient can detect the tactile perception. The test can reflect the peripheral sensory nerve function and involves the use of different size monofilaments to exert different amounts of pressure until the patient reports the tactile perceptions. The previous review reported the quantitative sensory testing (QST) had positive significant construct validity and sensitivity to detect true abnormalities. However, after discussing the results and limitations of the identified study, this previous review reported that any conclusions regarding the sensitivity and specificity of QST were probably inaccurate.

### Current perception threshold (CPT) test

CPT is a non-invasive method to detect the amplitude that a patient requires to detect a buzzing or tingling sensation through a neuro-meter. The previous systematic review indicates that this method can early detect periphery neuropathy[21].

### Wong-Baker FACES pain rating scale (WBS)

This scale contains six face drawings to assess the severity of pain in children [35]. The previous systematic review found that this scale was used to assess the neurological pain of children experiencing CIPN [21]. The scale is from 0 to 5, with “0” = no pain, “1” = slightly sore, “2” = slightly sore, “3” = sorer, “4” = very painful, and “5” = very painful, it hurts too much [35]. From the previous systematic review, the psychometric properties of the FACES scale have not been adequately examined and might not be feasible for use, particularly in young children (i.e., aged ≤ 5 years) [21].

### The mobility measures

The mobility measures to detect and diagnose CIPN in pediatric oncology patients included three-dimensional motion analysis (3-DMA), electromyography (EMG), goniometer assessment of passive ankle dorsiflexion range of motion, Medical Research Council guidelines for assessment of dorsi exor strength, plantar exor strength by the unipedal hopping test, and quantified gait characteristics [24]. From the previous review, the comprehensive mobility assessments might not be feasible for routine use in the pediatric population because specialized equipment or assessment skills are needed [21].

### Bruininks-Oseretsky test of motor proficiency version 2 (BOT-2)—balance subscale

The BOT-2 is designed to provide a comprehensive overview of fine and gross motor skills in children and young adults within school age-range [36]. The balance subscale contains nine items. Lower scores indicate increased impairment [36]. The previous systematic review did not report the psychometric properties of this subscale [21].

## Justification for an updated systematic review

Recently, the number of studies related to PROMs has exponentially increased. We also carried out a preliminary search to identify any PROMs of CIPN for children with cancer since 2018. The results of this preliminary search revealed some relevant PROMs, such as the Functional Assessment of Cancer Therapy-Gynecologic Oncology Group-Neurotoxicity (FACT-GOG-Ntx) [37]. Since 5 years have passed after publishing that systematic review, we aimed to update the systematic review of assessment tools on CIPN for pediatric oncology patients based on the evidence available after the published review in 2020.

## Methods

### Study design

This systematic review was an update of the previously published review [21] and adhered strictly to an updated guideline for reporting systematic review of Preferred Reporting Items for Systematic Reviews and Meta-analysis (PRISMA) [38]. The studies included in the previous review has summarized; therefore, only newly identified studies were analyzed. The same statistical methods, criteria for study eligibility, and quality assessment were used as in the previous review. The study protocol has already been registered at International Prospective Register of Systematic Reviews (PROSPERO) (reference number: CRD42024529326).

### Search strategy

We searched the PubMed, CINAHL, PsycINFO, EMBASE, the Cochrane Central Register of Controlled Trials, Scopus, and Web of Science for studies from Nov 9, 2018, to May 20, 2024. Searching keywords included but not limited to “chemotherapy,” “cancer,” “neoplasm,” “CIPN,” “peripheral,” “neurotoxicity,” “neuropathic pain,” “pediatric,” “adolescent,” and “children.” To select relevant studies, two authors (MT and FNY) separately screened the search results according to the eligibility criteria. The complete search strategies are presented in supplementary material 1. Any disagreement on the selection was resolved by discussion among the research team.

### Selection criteria

Tools were selected for inclusion if they met the following criteria: (1) study sample contained subjects who had a cancer diagnosis and were aged under 18 years according to the definition of child by United Nations International Children’s Emergency Fund (UNICEF) [39], (2) published in English language and peer-reviewed, (3) discussed the development of a tool to measure CIPN or accessed all test items and response categories for CIPN (e.g., items listed in factor analysis, or in the Appendix), (4) control groups were included if the groups exposed to neurotoxic agents could be analyzed independently, (5) was designed with the primary purpose of measuring CIPN, and not diabetic neuropathy or anticonvulsant drugs induced neurotoxicity or other conditions (e.g., radiation therapy-related neuropathy or stem-cell transplantation-related neuropathy), (6) the test was self-report or rated by healthcare professions, e.g., physicians, and (7) the tool was original and not an assessment component belonging to existing measures and/or other diagnostic criteria (e.g., Memorial Symptom Assessment Scale adapted to neurological symptoms). We excluded articles if they were animal studies or basic science research, were conference abstracts or proceedings, case studies, or expert opinions which did not include patients without a cancer diagnosis, did not describe patients who were not receiving or had not received chemotherapy, or were not designed to assess the psychometric properties of a pediatric CIPN instrument.

### Data extraction

The following information was extracted: author, year, study design, objective(s), sample, study setting, CIPN tools used, methods, cut-off value, reported psychometric properties including reliability, validity, sensitivity, responsiveness and feasibility, and limitations.

### Appraisal of study and instrument quality

The quality of each study was assessed based on Joanna Briggs Institute (JBI)’s critical appraisal tools for analytical cross-sectional studies and case control studies. They provide a standardized way to evaluate the extent to which a study has addressed the potential for bias in its design, methodology, and analysis. There were eight items to assess the study quality: (1) eligibility defined, (2) sample and setting described, (3) comparison group defined, (4) objective criteria for measuring condition, (5) adequate training of study staff, (6) attention to procedural fidelity, (7) strategies to consider confounders, and (8) appropriate statistics and statistical power. Each item can be rated as 0, 0.5, or 1. A score of 0 means that the characteristic is not shown by the study. A score of 1 means that the characteristic is shown by the study. A score of 0.5 means that the characteristic is partially shown by the study.

The quality of identified instruments for CIPN was evaluated by the modified version of Quality Assessment of Diagnostic Accuracy Studies (QUADAS) tool which was developed to evaluate the accuracy of diagnostic tests by evaluating their reliability and validity. This tool was adapted for application to measure and summarize the reliability and validity of CIPN measures. The QUADAS contains seven items, including whether the participants had CIPN, random selection, reliability estimates ≥ 0.70, evidence of construct validity, comparison reference standard, procedural detail, and instrument scoring procedures described. All items are dichotomous, with a score of 0 referring to “the characteristic is not shown” and a score of 1 as “the characteristic is shown.” The range of scores is 0–7, with scores of 0–3 indicating that the instrument having poor quality, 4–5 as moderate quality, and 6–7 as high quality.

Two reviewers (MT and QL) scored the studies independently according to the eight JBI characteristics, and three reviewers (MT, QL, and Getaneh) scored the tools independently based on the seven QUADAS characteristics. Any inconsistency of ratings was resolved in regular meetings led by a senior researcher (KYH).

### Synthesis method

We assessed each study using a narrative synthesis because the data was not appropriate for aggregation or meta-analysis because each assessment measure was found to have high heterogeneity for methodology.

### Psychometric evaluation

In our updated systematic review, we used the psychometric toolbox for testing reliability and validity that proposed by DeVon et al. [40] in 2007 to summarize the psychometric properties of the identified tools. Based on this guideline, the CIPN assessment tools were evaluated for their reported psychometric properties, including reliability, validity, sensitivity, responsiveness, and feasibility. Reliability was evaluated by the following indexes: internal consistency, test–retest reliability, alternative forms reliability, inter-rater reliability, and intra-rater reliability. Validity included construct validity, translational validity (face and content validity), and criterion validity (concurrent, predictive, convergent, discriminant validity). Sensitivity and responsiveness are also important measurement properties in addition to reliability and validity. Sensitivity is the ability of an instrument to detect and/or respond to subtle changes [41, 42]. Responsiveness is the ability of an instrument to measure a meaningful or important changes over time [41, 42]. Feasibility was defined as the time and resources required to collect and process the assessment, including ease of use, the need for staff training, and the time required to complete the assessment [43]. Supplementary file 2 shows the definition of each psychometric concept with its cut-off score in statistical analyses.

## Results

### Search results

**Fig. 1:**
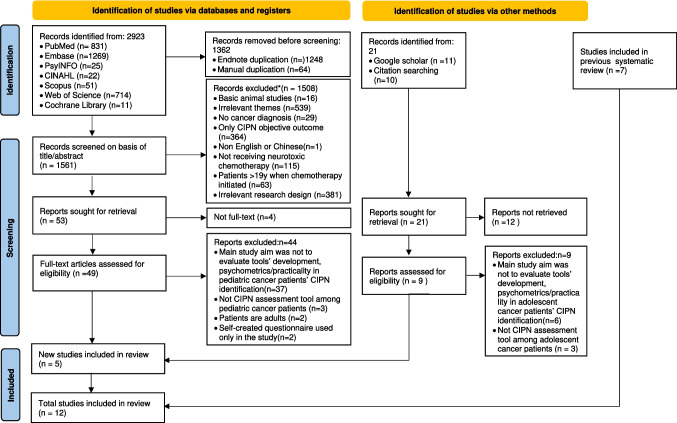
PRISMA 2020 flow chart

A total of 2923 articles were identified through the systematic search. After excluding 1362 duplicates, we screened the remaining 1561 articles for titles and abstracts, from which we retrieved 49 full-text reports. Nine full-text reports were additionally retrieved from 21 records from Google Scholar and references checking. After assessing for eligibility, 5 articles met the inclusion/exclusion criteria, which were added to the 7 studies previous identified. Thus, the total number of studies in this review was 12. The PRISMA flow diagram 2020 is shown in Fig. 1.

### Characteristics of newly included studies

A total of number of children of the newly included studies were 633. Excluding 96 healthy controls, 537 children with cancer were enrolled in the five papers, with the sample sizes ranging from 53 to 309. Concerning the patients’ demographic characteristics, four studies included children with age of 5–18 years [27, 44–46], and one study included pediatric oncology patients aged between 5 and 21.9 years [37]. 55.7% (*n* = 299) were male; 44.3% (*n* = 238) were female. For clinical characteristics, of the 537 children, 362 (67.4%) were actively receiving chemotherapy treatment [27, 37]; 56 (10.4%) were treated with at least four administrations chemotherapy within 6 weeks [46]; 40 (7.5%) were on chemotherapy for more than 2 months [45]; 79 (14.7%) were receiving or had received neurotoxic chemotherapy drugs at the time of the study [44]. Concerning the chemotherapy regimen, 72.2% (*n* = 388) were receiving or had received multiple neurotoxic chemotherapy drugs, e.g., vinca alkaloids, platinum, and cytotoxic drugs [37, 44]; 20.3% (*n* = 109) only receiving or treated with vincristine therapy [27, 46]; 7.5% (*n* = 40) were on vincristine and/or cisplatin therapy [45]. For tumor types, 57.5% (*n* = 309) were diagnosed with classic Hodgkin lymphoma [37]; 24.6% (*n* = 132) children were diagnosed with multiple cancer [27, 44]; 10.4% (*n* = 56) were pediatric oncology patients with non-central nervous system (CNS) malignancies [46]; 7.5% (*n* = 40) were diagnosed one of the cancers: leukemia, lymphoma, solid tumor [45]. Besides, one study was a longitudinal study [37], two studies were cross-sectional studies [27, 44], and two studies adopted a case–control design [45, 46]. The characteristics of each study are presented in Table 1.

**Table 1:** Summary of pediatric CIPN assessment measures evidence

Authors and date from the original publication	Study design and objective	Sample and setting	CIPN measures	Methods	Cut-off value	Reliability results	Validity results	Sensitivity, responsiveness, and feasibility results	Limitations
Susan K. Parsons, 2023 [1]	Design: Multi-center, descriptive, prospective longitudinal Objective: To assess the feasibility and psychometric performance of FACT-GOG-Ntx as part of a Phase 3 clinical trial	Children with cancer from USA, Each participating Children’s Oncology Group in North America: *N* = 309 - high risk Hodgkin lymphoma - 5–21.9 years: mean age = 14.99 years - 50.49% (*n* = 156) female, 49.51% (*n* = 153) male - are receiving 5 cycles of chemotherapy including a tubular inhibitor (vincristine), doxorubicin, bleomycin, etoposide, prednisone and cyclophosphamide (Arm 1: ABVE-PC), or the same chemotherapy backbone with the addition of BV and the absence of bleomycin (Arm 2: BV-AVE-PC)	FACT-GOG-Ntx	FACT-GOG-Ntx was collected serially prior to therapy initiation (Time 1), and on day 8 of cycles 2 (Time 2) and 5 (Time 3) Patients aged 5–17.9 years was assessed by their parent proxy raters, while patients aged 11–21.9 were only assessed by the youth themselves Internal consistency reliability: calculated with Cronbach’s alpha Intra-rater reliability: intraclass correlation coefficient Inter-rater reliability: intraclass correlation coefficient Construct validity: assessed with correlations between the FACT-GOG-Ntx, FACT-GOG-Ntx-4, and CHRIs-Global and comparing FACT-GOG-Ntx and FACT-GOG-Ntx-4 at Time 3 Responsiveness: assessed via a repeated measures model (proc mixed) of changes in β (SE) over time from baseline to Time 3 and effect size Feasibility: the percentage of CIPN assessments that were obtained in children 5–21.9 years of age	A change of 1/3 standard deviations (SD) on the total score and a 1-point change on the sensory subscale score was clinically significant	Internal consistency reliability: Cronbach’s alpha for FACT-GOG-Ntx Youth: 0.76 (baseline); 0.81(Time 2); 0.88 (Time 3); Cronbach’s alpha for FACT-GOG-Ntx Parent-proxy: 0.71 (baseline); 0.80 (Time 2); 0.90 (Time 3) Intra-rater reliability: The FACT-GOG-Ntx has acceptable intra-rater reliability based on an intraclass correlation coefficient (ICC) of 0.70 (95% CI 0.691–0.701) Inter-rater reliability: The FACT-GOG-Ntx has acceptable inter-rater reliability based on an ICC of 0.99 (95% CI 0.984–0.990)	Construct validity: CHRIs-Global measure for youth raters: 0.43 (baseline), 0.41 (Time 2), 0.45 (Time 3); CHRIs-Global measure for parent-proxy: 0.42 (baseline); 0.43 (Time 2); 0.48 (Time 3)	Responsiveness: - Youth FACT-GOG-Ntx was responsive to change based on statistically significant changes over time (*p* < 0.0001) and moderate SE (0.48) - Parent FACT-GOG-Ntx was responsive to change based on statistically significant changes over time (*p* < 0.0001) and moderate SE (0.45) Feasibility: - Youth & Parent FACT-GOG-Ntx attainable in 86.1% and 90.6% of children aged 11–21.9 years, 5 to 17.9 years, respectively	(1) Findings are only generalizable to patients with Hodgkin lymphoma who are receiving ABVE-PC and BV-AVE-PC (2) No control group (3) Fidelity procedures are not described
Ellen M. Lavoie Smith, 2021 [2]	Design: multi-center, prospective cross-sectional Objective: To evaluate sensitivity, internal consistency reliability, content and convergent validity, and clinical feasibility of P-CIN	Children with cancer from two academic sites: *N* = 79 - All diagnosis - 6–17 years: age = years −30 female, 49 male -Therapy: 100% were receiving or had received neurotoxic chemotherapy drugs (e.g., vinca alkaloids, platinums, taxanes, thalidomide, bortezomib)	P-CIN Ped-mTNS BOT ([2)	P-CIN: eight items were used to rate CIPN symptoms-numbness and tingling in the hands and feet-experienced today, five items were used to rate the child’s ability to perform a functional task that can be negatively influenced by CIPN by using a tablet computer. Examiners were blinded to P-CIN results Internal consistency reliability: Cronbach’s alpha and item–item correlations Content validity: a content validity index (CVI) was calculated using established techniques, in which five experts evaluated the survey’s content validity Construct validity: Convergent validity: assessed based on correlational analyses between the P-CIN score and scores from the ped-mTNS and the BOT Sensitivity: was determined using descriptive statistics based on whether the single item scores encompassed the full score range and whether floor or ceiling effects were demonstrated by item responses clustering together in high or low categories Clinical feasibility: assessed based on the percentage of children who could complete the measure independently	NR	Internal consistency reliability: the P-CIN demonstrated acceptable internal consistency with most items scoring range from 0.31 to 0.62 on the corrected item–item correlation and an overall Cronbach’s alpha of 0.86	Content validity: The overall content validity index coefficient was 1.0 (*p* = 0.05) and items ranged from 0.8 to 1.0 (*p* = 0.05) Construct validity: Convergent validity: P-CIN scores were strongly associated with ped-mTNS (*r* = 0.52, *p* < 0.01) and BOTMP (*r* = − 0.83, *p* = 0.04) scores	Sensitivity: Response ranges for toe numbness, pick up a coin (revised), and three of four pain items were 0 to 2, suggesting floor effects. The lowest item mean score was for No. 6 (revised; pick up a coin; *X* = 0.33, SD = 0.82, range = 0–2); the highest item mean score was for No. 7 (revised; standing on one leg; *X* = 2.67, SD = 1.21, range = 1–4) Clinical feasibility: 5 to 7 years could read and answer the questions independently. Children who needed assistance from a parent/guardian were younger than those who completed the measure independently	(1) Cross-sectional design prevented assessment of responsiveness to change (2) No control group (3) Nearly all received vincristine therapy (4) Three revised items (No. 6, 7, and 8) and BOT assessments were administered to a very small sample (*n* = 6)
Bilge Özdemir, 2023 [3]	Design: multi-site descriptive, prospective, cross-sectional study Purpose: To examine the Turkish validity and reliability of ssthe TNS-PV	Children with cancer from two university hospitals: *N* = 53 - All diagnosis, 81.8% of the children were diagnosed with ALL - 5–18 years: mean age = 9 ± 3.595 years −16 (30.2%) female, 37 (69.8%) male -Therapy: 100% were receiving vincristine therapy: dose received: 10.773 ± 6.600 mg	TNS-PV NCI-CTACE version 4.03 Wong-Baker FACES Pain Scale APPT	The TNS- PV scale evaluated numbness, tingling and neuropathic pain, proximal extension, vibration and warmth sensation, muscle strength, deep tendon reflexes, constipation, and hoarseness/vocal cord function. Scale items scored between 0 and 4. The signs and symptoms experienced in the hands versus the feet are not differentiated in the original TNS (Form A). An alternative TNS-PV scoring (Form B) was tested if the presence of both lower and upper extremity symptoms Internal consistency: Cronbach’s alpha coefficient and item-item correlations Inter-rater reliability: The Kappa coefficient Convergent validity: correlation between TNS-PV and scores on the NCI-CTCAE, APPT, and Wong-Baker FACES pain scale	NR	Internal consistency: Cronbach’s alpha value was found to be 0.628 over Form A and 0.639 over Form B Inter-rater reliability: TBS-PV Form A: A moderately positive and significant correlation was found between TNS-PV Form A total and worst subjective symptoms, strength, tendon reflexes, and autonomic/constipation (*r* = 0.441, *r* = 0.545, *r* = 0.472, *r* = 0.536, *p* < 0.01) A highly positive and significant correlation was found between TNS-PV Form A total and vibration sensitivity (*r* = 0.770, *p* < 0.01) TBS-PV Form B: A highly positive and significant correlation between Form B total and worst subjective symptom, vibration sensitivity, and autonomic/constipation (*r* = 0.648, *r* = 0.626, *r* = 0.635, *p* < 0.01) A moderately positive and significant correlation (*r* = 0.536, *r* = 599, *r* = 531, *r* = 0.482, *p* < 0.01) was found with temperature sensitivity, strength, tendon reflexes, and larynx/hoarseness	Convergent validity: A moderately positive and significant correlation was found between TNS-PV Form A total and APPT total percent score (*r* = 0.404, *p* < 0.05). A highly positive and significant correlation was found between TNS-PV Form A total and Wong-Baker FACES Pain scale (*r* = 0.608, *p* < 0.01) TNS-PV Form A and B total score was found to be correlated with NCI-CTCAE peripheral neuropathy rating scale and FACES pain scale. A moderately significant correlation was found between TNS-PV Form B total and NCI-CTCAE sensory neuropathy score and Wong-Baker FACES Pain scale (*r* = 0.503, *r* = 0.549, *p* < 0.01), and a highly positive and significant correlation was found with NCI-CTCAE motor neuropathy score (*r* = 0.695, *p* < 0.01)	NR	(1) 81.1% were diagnosed with ALL (2) Small sample size
S. M. Schouten, 2020 [4]	Design: multi-site, prospective, cross-sectional, case–control Purpose: To evaluate the construct validity and reliability of the Dutch version of the Pediatric-Modified Total Neuropathy Score (ped-mTNS) for assessing vincristine-induced peripheral neuropathy (VIPN) in Dutch pediatric oncology patients	Children with cancer from two university hospitals: *N* = 56 - non-central nervous maligancies - 5–18 years: median, IQR age = 9.6 (6.6–14.2) years −24 (42.9%) female, 32 (57.1%) male -Therapy: treated with at least four administrations of at least 1.5 mg/m^2^ (maximum 2 mg) VCR within a period of 6 weeks during treatment Age- and gender-matched healthy children: *N* = 56	Ped-mTNS NCI-CTCAE version 4.03	Ped-mTNS and NCI-CTCAE were performed by the same assessor, who was trained extensively by a pediatric neurologist to perform the VIPN assessments Intra-rater/inter-rater reliability: means of intra-class correlation coefficient using the two-way random effects model for agreement (ICC _agreement_) Construct validity: the correlation between the ped-mTNS and the NCI-CTCAE sum scores	NR	Intra-rater reliability: ICC_agreement_: 0.64 Inter-rater reliability: ICC_agreement_: 0.63	Construct validity: The correlation between total scores of the ped-mTNS and NCI-CTCAE was moderate (*r* = 0.60). Patients had a significantly higher score on ped-mTNS than healthy controls (median (IQR): 10.0 (6.25–13.0) and median (IQR): 0.0 (0.0–1.0), respectively; *p* < 0.001)	NR	(1) Participants received vincristine, limiting the generalizability (2) Small sample size
Bilge Özdemir, 2023 [5]	Design: multi-site, descriptive, prospective, cross-sectional, case–control Purpose: To perform the Turkish validity and reliability of Ped-mTNS	Children with cancer from two university hospital: *N* = 40 - Leukemia, lymphoma, solid tumor - 5–18 years: mean age = 9.7 ± 3.8 years −12 (30%) female, 28 (70%) male - On chemotherapy treatment (vincristine and/or cisplatin) for more than 2 months Age- and gender-matched healthy children: *N* = 40 - 9.1 ± 3.4 years - 12 (30%) female, 28 (70%) male	Ped-mTNS, NCI-CTCAE v4.0	Internal consistency reliability: Cronbach’s alpha and item-total score correlations Content validity: each item and the total content validity index Inter-rater reliability: intra-class correlation analysis Test–retest validity: intra-class correlations, an interval of 1 week Construct validity: Spearman correlation analysis	NR	Internal consistency reliability: the item-total correlations of the scale items ranged from 0.260 to 0.658 and an overall Cronbach’s alpha of 0.709 Inter-rater reliability: the inter-rater reliability of the ped-mTNS for the item and total scale scores was found to be acceptable (ICC > 0.95)	Content validity: inter-rater agreement was determined as 1.00 Test–retest validity: the Ped-mTNS total and intra-class correlation coefficients of the items ranged from 0.92 to 1.00 Construct validity: a significant positive correlation was found between the ped-mTNS total scale score and the evaluation of sensory neuropathy (*r* = 0.574, *p* < 0.001) and motor neuropathy (*r* = 0.645, *p* < 0.001) by NCI-CTCAE	NR	(1) Participants received vincristine and/or cisplatin, limiting the generalizability of the findings (2) Cross-sectional design (3) Limited assessment of sensitivity and specificity of individual items

*NA* not applicable, *FACT-GOG-Ntx* Functional Assessment of Cancer Therapy-Gynecologic Oncology Group-Neurotoxicity, *CHRIs-Global* Child Health Ratings Inventories-Global quality of life, *P-CIN* Pediatric Chemotherapy-Induced Neuropathy, *Ped-mTNS* Pediatric-modified Total Neuropathy Score, *BOT* Bruininks-Oseretsky Test of Motor Proficiency, *TNS-PV* Total Neuropathy Score-Paediatric Vincristine, *NCI-CTCAE* National Cancer Institute-Common Terminology Criteria for Adverse Events, *APPT* Adolescent Pediatric Pain Tool, *ICC* interclass correlation coefficients

### Characteristics of newly identified instruments

The newly identified instruments for CIPN included the following: (1) the Functional Assessment of Cancer Therapy-Gynecologic Oncology Group-Neurotoxicity (FACT-GOG-Ntx)[37], (2) the Pediatric Chemotherapy-Induced Neuropathy (P-CIN)[44], (3) Bruininks-Oseretsky Test of Motor Proficiency Second Edition short form (BOT-2 SF) [44], and (4) Adolescent Pediatric Pain Tool (APPT) [27]. Of them, the FACT-GOG-Ntx and P-CIN were PROMs. The BOT-2 SF was considered as an objective assessment. The APPT was a pain scale.

The total number of items in our newly identified instrument ranged from 3 to 14. All identified instruments were unidimensional except the APPT which was specified as multi-dimensional. The FACT-GOG-Ntx, P-CIN, and BOT-2 SF were available in English [37, 44], and APPT was available in Turkish version [27]. Items of the FACT-GOG-Ntx and P-CIN were evaluated on a Likert scale [37, 44], while the raw score of the BOT-2 SF was varied in items, ranging from 2 to 16 points [44]. The APPT contains three subscales, including pain locations ranged from 0 to 20, pain severity ranged from 0 to 10, and pain quality from 0 to 100%. The FACT-GOG-Ntx provided a cut-off for CIPN [27]. As for BOT-2 SF, the best cut-off point at was 13 [44]. No cut-off values reported for P-CIN and APPT [27, 44]. All instruments were described as appropriate for use by participants younger than 18 years. The minimum age of use for these four instruments was 5 [27, 37, 44–46]. Regarding application, all instruments were developed to be applied in both research and clinical settings [27, 37, 44–46].

### Narrative synthesis of the included studies

#### PROMs

##### The FACT-GOG-Ntx

Of our included studies, one reported the psychometric properties of the Functional Assessment of Cancer Therapy-Gynecologic Oncology Group-Neurotoxicity (FACT-GOG-Ntx) in children and adolescents with cancer. The FACT-GOG-Ntx has been widely used in adult cancer patients undergoing chemotherapy to provide comprehensive assessment of symptoms of peripheral neuropathy [47, 48], including numbness or tingling in hands/feet, discomfort in hands/feet, joint pain or muscle cramps, feeling weak all over, trouble hearing, ringing or buzzing in ears, trouble buttoning buttons, trouble feeling the shape of small objects in hand, and trouble walking within a 1-week interval [47, 48]. The FACT-GOG-Ntx is unidimensional and contains 11 items with each evaluated on a 5-point Likert scale (from 0 = “not at all” to 4 = “very much”). The range of scores is from 0 to 44; higher scores indicate worsen CIPN [47, 48].

Our identified study was conducted by Susan and colleagues [37] to assess the reliability, validity, and responsiveness of the FACT-GOG-Ntx in 309 children and adolescents with cancer (aged 5–21.9 years) from USA. Data collection was done in three different timepoints, including initiation of chemotherapy (Time 1), day 8 of 2nd cycle (Time 2), and 5 th cycle (Time 3) of the chemotherapy, resulting in 927 assessments [37]. Pediatric patients aged 5–10.9 years were assessed only by their parent proxy raters, while those aged 18–21.9 years were only assessed by themselves. For pediatric patients aged 11–17.9 years, they were assessed by themselves or their parent proxy raters [37]. However, the results from this group were not reported separately in terms of patient-report and proxy-report. Hence, we cannot compare how the children responded differently and the percentages of missing data by their ages. For the reliability, it was assessed in terms of internal consistency reliability, intra-rater reliability, and inter-rater reliability. The results revealed an acceptable internal consistency of the FACT-GOG-Ntx for self-reporting by adolescents, with the Cronbach’s alpha value of 0.76 (Time 1), 0.81(Time 2), and 0.88 (Time 3), and an acceptable internal consistency for proxy-report, with the Cronbach’s alpha value of 0.71 (Time 1), 0.80 (Time 2), and 0.90 (Time 3). Acceptable intra-rater (intraclass correlation coefficient 0.70, 95% CI 0.691–0.701) and excellent inter-rater (0.99, 0.984–0.990) reliability data were reported. Concerning validity, only construct validity was assessed, with the correlations between the FACT-GOG-Ntx and CHRIs-Global being calculated. Correlations between the scores of the FACT-GOG-Ntx and CHRIs-Global over the three time points for self-report by adolescents (0.41 to 0.45) and proxy-report by parents (0.42–0.48) were moderate. Construct validity was also supported by a comparison that patients who were clinically diagnosed to have CIPN (youth report: 39.34 ± 5.25/parent-proxy report: 39.26 ± 5.92) reported a statistically significantly higher mean score of the FACT-GOG-Ntx than those without CIPN (youth report: 32.98 ± 9.19/parent-proxy report: 32.80 ± 10.52) at Time 3. For responsiveness, a repeated measures model was used to assessing the change in FACT-GOG-Ntx scores over Time 1, 2, and 3 among child-parent dyads. The results showed that the FACT-GOG-Ntx scores at Time 3 were significantly lower for youth (*β* = − 2.83, *p* < 0.001) and parent-proxy raters (*β* = − 1.99, *p* < 0.001) when compared to baseline. This supported that the FACT-GOG-Ntx was highly responsive to change in CIPN symptoms over time. The results also demonstrated that older age in youth was associated with lower FACT-GOG-Ntx scores in parent-proxy raters (*β* = − 0.27, *p* = 0.01), but not in youth raters. This suggested that FACT-GOG-Ntx was more responsive to age-related changes in parent-proxy raters than in youth raters. For gender differences, the results showed that males had higher FACT-GOG-Ntx scores than females (*β* = 1.47, *p* < 0.001). This demonstrated that the FACT-GOG-Ntx was responsive to the influence of gender on the reporting of neurotoxicity symptoms.

##### P-CIN 

We identified one study which assessed another PROM named Pediatric Chemotherapy-Induced Neuropathy (P-CIN). It was originally developed for children ≥ 6 years old who had received or were receiving neurotoxic chemotherapy and reported peripheral neuropathy to quantify numbness and tingling in the hands and feet and the functional deficits in the past 2 to 3 days [44]. The P-CIN includes 13 items, with eight items to rate CIPN symptoms in the hands and feet and five items to rate the difficulty of performing functional tasks, e.g., standing on one leg and closing eyes for 15 s [44]. Each item is rated using a 6-point faces scale. The total score ranges from 0 to 65 with higher scores indicating more severe CIPN [44].

This identified study included 79 children aged 5 to 17 years old who had received or were receiving neurotoxic chemotherapy drugs, e.g., vinca alkaloids, platinums, taxanes, thalidomide, and bortezomib, to examine the psychometric properties of the P-CIN. The results showed that the P-CIN had acceptable internal consistency with the most items scorings ranging from 0.31 to 0.62 on the corrected item-item correlation and an overall Cronbach’s alpha of 0.86. Results of this study also supported that the P-CIN had strong content validity with the overall CVI coefficient of 1.0 and CVI coefficients for items ranging from 0.8 to 1.0. For convergent validity, the P-CIN scores were strongly associated with ped-mTNS (*r* = 0.52, *p* < 0.01) and BOT-2 SF (*r* = − 0.83, *p* = 0.04) scores, indicating a strong construct validity. This study also assessed the sensitivity of the P-CIN by examining the range of scores for each item to determine the presence of the floor and ceiling effects. The results illustrated floor effects for 5 items. Lastly, the clinical feasibility of the P-CIN was evaluated by calculating the percentage of children who could complete this scale without assistance. The P-CIN was deemed as a feasible tool with the completion rate ≥ 80% in clinical settings. However, in this study, only 68% of the participants could complete the P-CIN independently.

#### Objective assessment

##### BOT-2 SF

The BOT-2 SF (14 items) is used to measure motor skills in individuals aged 4 to 21 years [36]. It is categorized into four composite motor domains, with each containing two subscales, namely (1) fine manual control which includes fine motor precision and fine motor integration, (2) manual coordination which includes manual dexterity and upper-limb coordination, (3) body coordination which includes bilateral coordination and balance, and (4) strength and agility which includes running speed and agility and strength [36]. The total scores range from 0 to 88, which are then categorized and presented in standardized percentile in which ≥ 98% is described as well-above average, 84–97% as above average, 18–83% as the average, 3–17% as below average, and ≤ 2% as well-below average [36]. The time required to assess one individual using the BOT-2 SF varies between 15 and 20 min [49]. The scoring system varies with each item, ranging from a 2-point (pass/fail) to a 16-point scale. The raw score of each individual item is recorded in the unit measured (e.g., seconds, number of catches) and then converted into a numerical point score [36].

One newly identified study [44] also provided data related to the psychometric properties of the BOT-2 SF for CIPN in pediatric oncology patients. One study of these studies [44] adopted the BOT-2 SF to objectively evaluate CIPN among children with cancer and used it as an accurate measure to determine the construct validity of the P-CIN, a PROM measure of CIPN.

#### Pain tools

##### APPT

In the literature search, we identified a new assessment tool to assess pain severity due to peripheral neuropathy. This new assessment tool is Adolescent Pediatric Pain Tool (APPT)[27]. The APPT is a self-reported, multidimensional measure of pain for children and adolescents between 8 and 17 years old [50]. The APPT provides three subscale scores: (1) pain location: measured by marking on the non-gender, front and back views of the body outline which are divided in 43 different locations; (2) pain intensity: measured by a 100-mm line known as the Word Graphic Rating Scale (WGRS) with different anchors representing “no pain,” “little pain,” “medium pain,” “large pain,” and “worst possible pain”; (3) pain quality: a list of 67 pain quality descriptors to evaluate four domains of pain, that is 37 items for sensory subscale (e.g., “like an ache,” “like a hurt”),11 items for affective subscale (e.g., “deadly,” “frightening”), 8 items for evaluative subscale (e.g., “bad,” “miserable”), and 11 items for temporal subscale (e.g., “always,” “once in a while”) [50].

The construct validity of APPT in children with cancer was reported by the study which primarily aimed to examine the Turkish psychometric properties of the TNS-PV to measure CIPN among children with cancer aged 5–18 years [27]. The mean score of the TNS-PV Form A total was found to have a moderate positive and significant correlation with the APPT total score (*r* = 0.404, *p* < 0.05).

Apart from the newly identified tools for CIPN, in the newly included studies, psychometric properties of the identified scales in the previous systematic review have been extracted and summarized.

##### Ped-mTNS 

We identified two new studies which validated ped-mTNS in Dutch [46] and Turkish [45] versions. These two studies provided data of reliability and validity of these two language versions. One study was conducted in two university hospitals, with 56 children with non-central nervous malignancies and 56 age- and gender-matched healthy children to examine the inter-rater reliability, intra-rater reliability, and construct validity of the Dutch version of the ped-mTNS [46]. Results from the two-way random effects model for agreement indicated moderate level for inter-rater reliability (inter-class correlation coefficient = 0.63; standard error of measurement = 3.7) and intra-rater reliability (intra-class correlation coefficient = 0.64; standard error of measurement = 2.92) [46]. The construct validity of the Dutch version was determined by calculating the correlation between the ped-mTNS and the NCI-CTCAE version 4.03 sum scores in patients as well as the differences between the median ped-mTNS scores reported by patients and their healthy controls. The correlation between the two scores was moderate (*r* = 0.60). Patients were found to have a statistically significantly higher median ped-mTNS score than healthy controls [46]. Another study was conducted in 40 children (mean age, 9.7 ± 3.8 years) with leukemia, lymphoma, and solid tumor to examine the inter-rater reliability, internal consistency reliability, test–retest reliability, and construct validity of the Turkish version [45]. Acceptable inter-rater reliability (ICC > 0.95 for the item and total scale scores) was found. Internal consistency reliability was evaluated by Cronbach’s alpha and item-total correlations. Adequate internal consistency (Cronbach’s alpha of 0.709) and acceptable item-total correlations (range from 0.260 to 0.658) were found. For test–retest reliability, 10 children were chosen to complete the ped-mTNS before and after a 1-week interval. The test–retest reliability was excellent as the intra-class correlation coefficients of the items between the one-week interval ranged from 0.92 to 1.00. The construct validity was also supported by a positive correlation between the ped-mTNS total score and the degrees of sensory neuropathy (*r* = 0.574, *p* < 0.001) and motor neuropathy (*r* = 0.645, *p* < 0.001) as measured by NCI-CTCAE version 4.0 in patients. Concerning sensitivity and clinical feasibility of the ped-mTNS, they were not assessed in these two studies.

##### TNS-PV

A new study [27] was identified to provide additional information of the psychometric properties of the Total Neuropathy Score-Pediatric Vincristine (TNS-PV). Our identified study was conducted by Bilge et al. to assess the internal consistency, inter-rater reliability, and convergent validity of the TNS-PV in 53 children aged 5–17 years who received Vincristine in the treatment [27]. In this study, the Cronbach’s *α* coefficient was used to calculate the internal consistency of the TNS-PV. The results showed that the TNS-PV had a moderate level of internal consistency (Cronbach’s *α* value of 0.628 for TNS-PV Form A; Cronbach’s *α* value of 0.639 for TNS-PV Form B). In addition, the Kappa coefficient was evaluated for inter-rater reliability [51]. The results showed a low-to-high inter-rater reliability (low: *r* = 0.324, *p* < 0.05/*r* = 0.398, *p* < 0.01; moderate: *r* = 0.441, *r* = 0.545, *r* = 0.472, *r* = 0.536, *p* < 0.01; high: *r* = 0.770, *p* < 0.01) for the TNS-PV Form A and a moderate-to-high inter-rater reliability (moderate: *r* = 0.648, *r* = 0.626, *r* = 0.635, *p* < 0.01; high: *r* = 0.536, *r* = 0.599, *r* = 0.531, *r* = 0.482, *p* < 0.01) for the TNS-PV Form B. Concerning convergent validity, the correlation between scores of TNS-PV and NCI-CTCAE, APPT, and Wong-Baker FACES Pain scales was evaluated. The highly positive and moderately positive correlations were found between TNS-PV Form A total scores and Wong-Baker FACES Pain scale score (*r* = 0.608, *p* < 0.01) and between TNS-PV Form A total scores and APPT total percent score (*r* = 0.404, *p* < 0.05), respectively.

##### NCI-CTCAE

We identified three new studies [27, 45, 46] which provided data related to the psychometric properties of the NCI-CTCAE grading scale. One study published by Bilge and colleagues [27] assessed the convergent validity of the NCI-CTCAE version 4.03 via analyzing the correlations between the scores of the NCI-CTCAE and TNS-PV. A moderately significant correlation was found between the TNS-PV Form B total scores and the NCI-CTCAE sensory neuropathy scores (*r* = 0.503, *p* < 0.01). In addition, a high correlation was found between the TNS-PV Form B total scores and the NCI-CTCAE motor neuropathy scores (*r* = 0.695, *p* < 0.01). Other two studies similarly examined the construct validity of the NCI-CTCAE via calculating the correlation between the NCI-CTCAE scores and the ped-mTNS scores [45, 46]. A significant correlation was found between the ped-mTNS total scale scores and the scores of sensory neuropathy (*r* = 0.574, *p* < 0.001) and motor neuropathy (*r* = 0.645, *p* < 0.001) by NCI-CTCAE among 40 children with cancer aged between 5 and 18 years [45]. The same result was also found in Schouten’s study [46] in which the correlation between total scores of the ped-mTNS and NCI-CTCAE version 4.03 was moderate in 56 children with non-central nervous system cancer aged 5–18 years (*r* = 0.60).

##### The Wong-Baker FACES Pain scale

We also found one study [27] which provided additional information about the construct validity of the Wong-Baker FACES Pain Scale which was the identified measure for CIPN in previous systematic review. This study showed that the Wong-Baker FACES Pain Scale scores were highly correlated (*r* = 0.608, *p* < 0.01) with the TNS-PV Form A scores and were moderately correlated (*r* = 0.549, *p* < 0.01) with the TNS-PV Form B scores.

### Quality assessment

#### Quality of included studies

We assessed the quality of included five studies based on the JBI (Table 2). Of the five included studies, only one was rated as high quality [44]. The remaining were rated as low to moderate quality [27, 37, 45, 46]. Most studies did not define the comparison group, not explain whether the assessment staff received the adequate training, not mention the procedural fidelity, and not involved the strategies to consider confounders[27, 37, 45, 46].

**Table 2:** Critical appraisal of articles reviewed by use of the JBI recommendations

Author	CIPN measures tested	Eligibility defined	Sample and setting described	Comparison groups defined	Objective criteria for measuring condition	Adequate training of study staff	Attention to procedural fidelity	Strategies to consider confounders	Appropriate statistics and statistical power	Total score
Parsons et al. (2023) [1]	FACT-GOG-Ntx	1.0	1.0	0.0	0.0	0.0	0.5	0.0	1.0	3.5
Smith et al. (2021) [2]	P-CIN, Ped-mTNS, BOT-2 SF	1.0	1.0	0.0	1.0	0.5	1.0	0.5	1.0	6.0
Özdemir et al. (2023) [3]	TNS-PV, NCI-CTACE version 4.03, Wong-Baker FACES Pain Scale, APPT	1.0	1.0	0.0	1.0	1.0	0.0	0.5	0.0	4.5
Schouten et al. (2020) [4]	Ped-mTNS, NCI-CTCAE	1.0	1.0	1.0	1.0	1.0	0.0	0.5	0.0	5.5
Özdemir et al. (2023) [5]	Ped-mTNS, NCI-CTCAE v4.0	1.0	1.0	1.0	1.0	0.0	0.0	0.0	0.5	4.5

A score of 0 means that the characteristic is not shown by the study. A score of 1 means that the characteristic is shown by the study. A score of 0.5 means that the characteristic is partially shown by the study. *JBI* Joanna Briggs Institute, *CIPN* chemotherapy-induced peripheral neurotoxicity, *FACT-GOG-Ntx* Functional Assessment of Cancer Therapy-Gynecologic Oncology Group-Neurotoxicity, *P-CIN* Pediatric chemotherapy-induced neuropathy, *Ped-mTNS* Pediatric-Modified Total Neuropathy Score, *BOT-SF* Bruininks-Oseretsky Test of Motor Proficiency short form, *TNS-PV* Total Neuropathy Score-Pediatric Vincristine, *NCI-CTCAE* National Cancer Institute Common Terminology Criteria for Adverse Events, *NA* not applicable, *APPT* Adolescent Pediatric Pain Tool

#### Quality of assessment tools for CIPN

We assessed the quality of identified assessment tools by using the QUADAS (Table 3). The overall quality of the included assessment tools ranged from moderate to high quality. The P-CIN was ranked with the best quality, with the QUADAS score of 6 [44]. The second was the ped-mTNS, with the QUADAS score of 5 based on two studies [45, 46]. The TNS-PV [27] and FACT-GOG-Ntx [37] were both ranked as the third, with the QUADAS score of 4.

**Table 3:** Critical appraisal of articles reviewed by use of the QUADAS assessment method

Author	CIPN measures tested	Participants had CIPN	Random selection	Reliability estimates ≥ 0·70	Evidence of construct validity	Comparison to reference standard	Procedural detail	Instrument scoring procedures described	Total QUADAS score
Parsons et al. (2023) [1]	FACT-GOG-Ntx	0.0	1.0	1.0	1.0	0.0	1.0	0.0	4.0
Smith et al. (2021) [2]	P-CIN, Ped-mTNS, BOT-2 SF	1.0	1.0	0.0	1.0	1.0	1.0	1.0	6.0
Özdemir et al. (2023) [3]	TNS-PV, NCI-CTACE version 4.03, Wong-Baker FACES Pain Scale, APPT	0.0	0.0	0.0	1.0	1.0	1.0	1.0	4.0
Schouten et al. (2020) [4]	Ped-mTNS, NCI-CTCAE	0.0	1.0	0.0	1.0	1.0	1.0	1.0	5.0
Özdemir et al. (2023) [5]	Ped-mTNS, NCI-CTCAE v4.0	0.0	0.0	1.0	1.0	1.0	1.0	1.0	5.0

A score of 0 means that the characteristic is not shown by the study. A score of 1 means that the characteristic is shown by the study. Scores reflect the mean of 3 scores from three independent reviewers. *QUADAS* Quality Assessment of Diagnostic Accuracy Studies, *CIPN* chemotherapy-induced peripheral neurotoxicity, *FACT-GOG-Ntx* Functional Assessment of Cancer Therapy-Gynecologic Oncology Group-Neurotoxicity, *P-CIN* Pediatric chemotherapy-induced neuropathy, *Ped-mTNS* Pediatric-Modified Total Neuropathy Score, *BOT-SF* Bruininks-Oseretsky Test of Motor Proficiency short form, *TNS-PV* Total Neuropathy Score-Pediatric Vincristine, *NCI-CTCAE* National Cancer Institute Common Terminology Criteria for Adverse Events, *APPT* Adolescent Pediatric Pain Tool

## Discussion

The systematic review aimed to update the available assessment tools for CIPN among pediatric oncology patients. We newly identified four tools including two PROMs that can be applied to capture the experience of CIPN from patients’ perspective, which address a major key concern identified in the previous systematic review.

In this updated systematic review, we identified a new PROM named the FACT-GOG-Ntx that can be used to assess CIPN in childhood cancer patients [37]. The evidence from included studies supported it as a valid and reliable instrument with the QUADAS score of 4 in our quality assessment. This PROM contains some distinctive advantages. Firstly, patients can easily complete the instruments within 10–15 min [48, 52] which is feasible in busy clinical settings. This is supported by a high completion rate of more than 90% in our included studies [37]. Importantly, findings from our included studies supported that this questionnaire can be completed by pediatric oncology patients themselves and their parents, with moderate-to-high agreement between the two ratings [37]. This provides an alternative option for healthcare professionals to assess the patients’ symptom experience when the patients are unable to report the experience themselves. Despite our findings support the use of the FACT-GOG-Ntx as a PROM in clinical settings, it contains a limitation in which this scale was only validated in pediatric oncology patients with Hodgkin lymphoma who were receiving vincristine, doxorubicin, bleomycin, etoposide, prednisone, and cyclophosphamide as their chemotherapy. The psychometric properties of the FACT-GOG-Ntx to assess CIPN for pediatric oncology patients with other diagnosis or receiving other chemotherapeutic agents remain uncertain. More studies are required to examine the psychometric properties of the FACT-GOG-ntx among pediatric oncology patients in more diverse clinical background. However, the identified study did not report whether there was any item that could not be answered by children. Hence, more studies should be conducted to determine whether all items are easily understandable by children. If not, amendment should be made to enhance the readability of specific items.

Another new PROM is the P-CIN which is the first assessment tool specifically developed for pediatric oncology patients to assess their CIPN [44]. According to our evaluation using QUADS, it received a score of 6.0 which is higher than that for the FACT-GOG-Ntx. The result suggested that the P-CIN is a tool with better psychometric properties than the FACT-GOG-Ntx to measure CIPN in pediatric oncology patients. Apart from the psychometric properties, there are some advantages of the P-CIN. Firstly, it can be used by a wide age range of children, starting from 6 to 18 years old [44]. It allows children who are young to report their CIPN experience notwithstanding their limited language proficiency [44]. Secondly, the P-CIN was developed to be administered using a tablet computer, which is in line with the current development in cancer care, that is to collect PROMs using an electronic mean and the collected information can be later incorporated into patients’ electronic medical records to guide clinical decision making [53, 54]. Despite these advantages, the feasibility of the P-CIN in pediatric oncology patients warrants our attention because a previous study showed that only 68% of the participants could complete the P-CIN without adult assistance [44]. Future studies are required to explore the reasons; amendment and more precise instructions prior to the survey can then be made to improve the feasibility of the P-CIN among pediatric oncology patients. Another issue is the floor effect in which a majority of the participants rated very low for some items assessing pain, numbness in toes, and capability to pick up a coin [44]. This compromised the sensitivity of the P-CIN to differentiate among pediatric oncology patients with low levels of CIPN. Also, the psychometric properties of the P-CIN were only examined in one study which were predominated by participants (63%) with lymphoid leukemia. In addition, 91.1% of the participants were receiving vincristine in their chemotherapy [44]. Since CIPN is dependent on cancer diagnosis and importantly neurotoxic agent, more research is needed to thorough examine the psychometric properties of the P-CIN. Besides, the P-CIN is now only available in English version. Translation works are necessary to expand the use of the P-CIN in other countries.

Apart from the PROM, this systematic review also identified two new instruments of CIPN for pediatric oncology patients. One is the BOT-2 SF which is an objective measure. In the systematic search, we only identified one study which demonstrated the construct validity of the BOT-2 SF in measuring CIPN [44]. Other psychometric properties, including internal consistency, test-re-test reliability, inter-rater agreement, content validity, criterion validity, and sensitivity responsiveness remain uncertain. Likewise, the BOT-2 SF was primary developed to assess the motor function [36] which is only one of the aspects of CIPN [7]. Other major aspects, such as sensory and autonomic symptoms [7] were not assessed. Also, the completion time for the BOT-2 SF is long which takes 20 min and demands the assistance of trained professionals to perform the assessment [36]. As such, the appropriateness of the BOT-2 SF in assessing CIPN among pediatric oncology patients is doubtful.

Another new instrument identified in our systematic search is the APPT. Similar to the Wong-Baker FACES Pain Scale, the APPT was applied to assess the pain associated with neuropathy among pediatric oncology patients [55]. When compared to the Wong-Baker FACES Pain Scale, the APPT appears to be more comprehensive as it captures pain associated with neuropathy in terms of location, intensity, and quality [35, 56]. However, existing literature only provided some support on its construct validity to assess CIPN, but not other psychometric properties. In addition, it is unable to capture all aspects of CIPN [35]. Hence, the APPT is not a stand-alone measure of CIPN for pediatric oncology patients.

We also identified new studies which provided supplementary information for the previously identified tools which assess CIPN. Concerning the Ped-mTNS which was suggested to be a moderately reliable and valid instrument [21], two more studies were found to translate the Ped-mTNS into two different versions which expands its coverage for pediatric oncology patients in different cultural origins [45, 46]. Consistent with the previous systematic review, these two studies provide moderate evidence to support the reliability and validity of the validated versions [45, 46]. However, these two studies did not address the floor effect and clinical feasibility of the Ped-mTNS which were emphasized in the previous systematic review. Hence, the Ped-mTNS still cannot be considered as a gold standard measure for pediatric CIPN. As for the TNS-PV, we identified one study which translated it into Turkish and validated in children with cancer who received vincristine as the chemotherapy [27]. This study adopted a prospective approach which addressed a major limitation in the previous study that collected data retrospectively [18]. Notwithstanding the limitation has been addressed, the evidence from our newly identified study was only moderate which is different from the previous review that provided high quality of evidence to support the psychometric properties of the TNS-PV. This is because, the newly identified study did not examine the test–retest reliability. The Cronbach’s alpha coefficient was less than 0.7 [27], which is lower than the Cronbach’s alpha of 0.84 in the previous study [18]. The newly identified study also did not examine the sensitivity, responsiveness, and feasibility of the Turkish version [27]. These limitations shall be further addressed and examined in future studies.

Our newly identified studies provided evidence on the construct validity of the Wong-Baker FACES Pain Scale [27] and NCI-CTACE [27, 45, 46] which is in contrast with the previous systematic review [21]. This can be attributed to the differences in study samples and improved methodology [21, 27, 45, 46]. Although the construct validity was supported, there was no other psychometric evaluation in the newly included studies. As such, the sensitivity issue identified by the previous systematic review is still a concern for NCI-CTCAE, while there is still inadequate evidence regarding the psychometric properties, e.g., reliability, sensitivity, responsiveness, and feasibility of the Wong-Baker FACES Pain Scale. Therefore, these two instruments are not recommended to be used in clinical settings to assess CIPN in pediatric oncology patients.

One worth noting thing is the recent development of the Pediatric Patient-Reported Outcomes version of the Common Terminology Criteria for Adverse Events (Ped-PRO-CTCAE)[57] which is a pediatric module of PRO-CTCAE to report the symptoms of pediatric patients without the involvement of clinician’s assessments. Ped-PRO-CTCAE includes self-report by children patients aged 7–17 years and proxy-report by caregivers for children aged 7–17 years who are unable to self-report. Under the Ped-PRO-CTCAE, there are also a subset of questions, such as numbness and tingling, muscle weakness, and muscle pain [58] for CIPN symptoms which can be a potential PROM to assess CIPN among pediatric oncology patients with appropriate psychometric evaluation. Ped-PRO-CTCAE is now being evaluated by multiple stakeholders and is considered a promising tool to provide a standard method to assess symptomatic adverse events from the patient perspective.

## Implication for clinical practice

CIPN is a devastating symptom which requires clinical attention and timely intervention. In this updated systematic review, we identified four additional instruments for CIPN and more evidence on the psychometric properties of existing instruments. Based on the current evidence, the Ped-mTNS and TNS-PV are still the two most appropriate tools for healthcare professionals to use in clinical settings. Importantly, our updated systematic review identified two PROMs for CIPN among pediatric oncology patients which bridged an important gap in existing literature. The quality of these two PROMs is acceptable and one PROM is high quality and hence can be applied to directly capture the patients’ experience in CIPN. These two PROMs have their own advantages and disadvantages. The selection among these two should be based on the clinical and research needs. In fact, an increasing number of evidences has suggested that the assessment of physician is unable to capture all patients’ experience [59], with more than 50% of symptomatology being overlooked [60]. This pointed out the importance and necessities to combine both PROMs and physician-based assessment tools to guide the treatment. The findings of our updated systematic review facilitate the integration of these two assessment approaches in pediatric oncology settings.

## Limitations

Despite the strengths and the important findings, this systematic review contains some limitations. One of the limitations is that we only included the studies published in English. Hence, studies published in other languages were excluded and we might not be able to cover all assessment tools for CIPN in pediatric oncology patients. Another limitation is that the literature search was done in the commonly used databases, e.g., PubMed. Some relevant literature in other databases might be missed in this updated systematic review.

## Conclusion

This updated systematic review identified four additional assessment tools for CIPN in pediatric oncology patients as well as more evidence to supplement the psychometric properties of identified assessment tools. Concerning the physician-based assessment tools, the Ped-mTNS and TNS-PV were found to be the appropriate tools that can be used to assess CIPN for this population group in clinical settings. Our results also addressed the gap in existing literature by showing two newly PROMs for CIPN in pediatric oncology patients, that is, FACT-GOG-Ntx and P-CIN. The combination of use of physician-based assessment tools and PROMs are recommended to thoroughly capture the patients’ symptom experience.

## Supplementary Information

Below is the link to the electronic supplementary material.

Supplementary file1 (DOCX 50 KB)

## Data Availability

No datasets were generated or analysed during the current study.

## Declarations

### Patient and public involvement

This study does not involve human participants.
