# Supplementary material for: Assessment measures for chemotherapy-induced peripheral neuropathy among pediatric oncology patients: an updated systematic review
Source: Support Care Cancer. 2025 May 29;33(6):514. doi: 10.1007/s00520-025-09515-5 (PMC12122654; doi:10.1007/s00520-025-09515-5)
Supplement: Supplementary file 1 — Supplementary file1 (DOCX 50 KB) [file 520_2025_9515_MOESM1_ESM.docx]

# **Appendix 1 Search Strategies**

1. **Pubmed (1976-, Search on 30, June, 2024)**

| **Concept** | **Search terms** | **n** |
| --- | --- | --- |
| #1 | Peripheral Nervous System Diseases/chemically induced[MeSH Terms] OR peripheral neuropathy[Title/Abstract] OR neuropathic[Title/Abstract] OR neurotoxicity[Title/Abstract] OR chemotherapy induced peripheral neuropathy[Title/Abstract] | 107,553 |
| #2 | (chemotherapy[Title/Abstract]) OR (chemotherapies[Title/Abstract]) OR (vincristine[Title/Abstract]) OR (oncovin[Title/Abstract]) OR (vinblastine[Title/Abstract]) OR (vinorelbine[Title/Abstract]) OR (vindesine[Title/Abstract]) OR (vinorelbine[Title/Abstract]) OR (vinca alkaloid[Title/Abstract]) OR (vinca alkaloids[Title/Abstract]) OR (oxaliplatin[Title/Abstract]) OR (eloxatin[Title/Abstract]) OR (cisplatin[Title/Abstract]) OR (carboplatin[Title/Abstract]) OR (platinum[Title/Abstract]) OR (platinums[Title/Abstract]) OR (taxane[Title/Abstract]) OR (taxanes[Title/Abstract]) OR (docetaxel[Title/Abstract]) OR (paclitaxel[Title/Abstract]) OR (ifosfamide[Title/Abstract]) OR (ifex[Title/Abstract]) OR (Ixabepilone[Title/Abstract]) OR (epothilone[Title/Abstract]) OR (Epothilones[Title/Abstract]) OR (Bortezomib[Title/Abstract]) OR (proteasome inhibitor[Title/Abstract]) OR (proteasome inhibitors[Title/Abstract]) OR (Thalidomide[Title/Abstract]) OR (Lenalidomide[Title/Abstract]) OR (procarbazine[Title/Abstract]) OR (thiotepa[Title/Abstract]) OR (podophyllin[Title/Abstract]) OR (topoisomerase inhibitor[Title/Abstract]) OR (teniposide[Title/Abstract]) OR (etoposide[Title/Abstract]) OR (vepesid[Title/Abstract]) OR (gemcitabine[Title/Abstract]) OR (Induction Chemotherapy[MeSH Terms]) OR (chemotherapy, adjuvant[MeSH Terms]) OR (Consolidation Chemotherapy[MeSH Terms]) OR (Maintenance Chemotherapy[MeSH Terms]) OR (Induction Chemotherapy[Title/Abstract]) OR (Chemotherapy, Adjuvant[Title/Abstract]) OR (Consolidation Chemotherapy[Title/Abstract]) OR (Maintenance Chemotherapy[Title/Abstract]) | 650,906 |
| #3 | ("cancer"[Title/Abstract] OR "oncology"[Title/Abstract] OR "tumor"[Title/Abstract] OR "tumour"[Title/Abstract] OR "carcinoma"[Title/Abstract] OR "malignancy"[Title/Abstract] OR "malignancies"[Title/Abstract] OR "malignant"[Title/Abstract] OR "neoplas*"[Title/Abstract] OR "Neoplasms"[MeSH Terms] OR "carcinoma"[MeSH Terms] OR "Neoplasms"[Title/Abstract] OR "carcinoma"[Title/Abstract] OR "glioma*"[Title/Abstract] OR "lymphoma*"[Title/Abstract] OR "myeloma*"[Title/Abstract] OR "leukemia*"[Title/Abstract] OR "leucaemia*"[Title/Abstract]) | 5,326,408 |
| #4 | ("child"[MeSH Terms] OR "child*"[Title/Abstract] OR "pediatrics"[MeSH Terms] OR "pediatric*"[Title/Abstract] OR "paediatric*"[Title/Abstract] OR "Adolescent"[MeSH Terms] OR "Adolescent"[Title/Abstract] OR "Adolescence"[Title/Abstract] OR "Teen"[Title/Abstract] OR "teenager"[Title/Abstract] OR "teens"[Title/Abstract] OR "Minors"[Title/Abstract] OR "Minors"[MeSH Terms] OR "youth"[Title/Abstract] OR "young adult"[Title/Abstract] OR "young adult"[MeSH Terms]) | 4,574,878 |
| #5 | (evaluation*[Title/Abstract] OR assessment*[Title/Abstract] OR psychometric*[Title/Abstract] OR measure*[Title/Abstract] OR propert*[Title/Abstract] OR develop*[Title/Abstract] OR reliab*[Title/Abstract] OR valid*[Title/Abstract] OR responsive*[Title/Abstract] OR method*[Title/Abstract] OR tool*[Title/Abstract] OR instrument*[Title/Abstract] OR scale*[Title/Abstract] OR survey*[Title/Abstract] OR questionnaire*[Title/Abstract] OR version*[Title/Abstract] OR checklist*[Title/Abstract] OR indicator*[Title/Abstract] OR profile*[Title/Abstract] OR index*[Title/Abstract] OR indices*[Title/Abstract] OR 'self report'[Title/Abstract]) | [17,632,526](https://pubmed.ncbi.nlm.nih.gov/?term=evaluation%2A%5BTitle%2FAbstract%5D+OR+assessment%2A%5BTitle%2FAbstract%5D+OR+psychometric%2A%5BTitle%2FAbstract%5D+OR+measure%2A%5BTitle%2FAbstract%5D+OR+propert%2A%5BTitle%2FAbstract%5D+OR+develop%2A%5BTitle%2FAbstract%5D+OR+reliab%2A%5BTitle%2FAbstract%5D+OR+valid%2A%5BTitle%2FAbstract%5D+OR+responsive%2A%5BTitle%2FAbstract%5D+OR+method%2A%5BTitle%2FAbstract%5D+OR+tool%2A%5BTitle%2FAbstract%5D+OR+instrument%2A%5BTitle%2FAbstract%5D+OR+scale%2A%5BTitle%2FAbstract%5D+OR+survey%2A%5BTitle%2FAbstract%5D+OR+questionnaire%2A%5BTitle%2FAbstract%5D+OR+version%2A%5BTitle%2FAbstract%5D+OR+checklist%2A%5BTitle%2FAbstract%5D+OR+indicator%2A%5BTitle%2FAbstract%5D+OR+profile%2A%5BTitle%2FAbstract%5D+OR+index%2A%5BTitle%2FAbstract%5D+OR+indices%2A%5BTitle%2FAbstract%5D+OR+%27self+report%27%5BTitle%2FAbstract%5D&sort=relevance) |
| #6 | #1 and #2 and #3 AND #4 AND #5 | 831 |

1. **Embase (OVID) (1910-, Search on 20, March, 2024)**

| **Concept** | **Search terms** | **n** |
| --- | --- | --- |
| #1 | ('peripheral neuropathy'/exp OR 'neuropathy, peripheral':ab,ti OR 'peripheral nerve disease':ab,ti OR 'peripheral nerve disorder':ab,ti OR 'peripheral nervous disease':ab,ti OR 'peripheral nervous system diseases':ab,ti OR 'peripheral neuropathy':ab,ti OR 'peripheral neurotoxicity'/exp OR 'neurotoxicity'/exp OR 'neurotoxia':ab,ti OR 'neurotoxic activity':ab,ti OR 'neurotoxicosis':ab,ti OR 'neurotoxicity':ab,ti OR 'chemotherapy-induced peripheral neuropathy':ab,ti OR 'chemotherapy-induced peripheral neuropathy'/exp) | 232,896 |
| #2 | ('chemotherapy'/exp OR 'chemotherapeutics':ab,ti OR 'chemotherapy':ab,ti OR 'vincristine'/exp OR vincristine:ab,ti OR vinblastine:ab,ti OR 'vinblastine'/exp OR 'vinorelbine tartrate'/exp OR 'vinorelbine tartrate':ab,ti OR 'vindesine'/exp OR vindesine:ab,ti OR 'vinca alkaloid'/exp OR 'vinca alkaloid':ab,ti OR 'oxaliplatin'/exp OR oxaliplatin:ab,ti OR 'cisplatin'/exp OR cisplatin:ab,ti OR 'carboplatin'/exp OR carboplatin:ab,ti OR 'platinum'/exp OR platinum:ab,ti OR 'taxane'/exp OR taxane:ab,ti OR 'docetaxel'/exp OR docetaxel:ab,ti OR 'paclitaxel'/exp OR paclitaxel:ab,ti OR 'ifosfamide'/exp OR ifosfamide:ab,ti OR 'thalidomide'/exp OR thalidomide:ab,ti OR 'etoposide'/exp OR etoposide:ab,ti OR 'gemcitabine'/exp OR gemcitabine:ab,ti OR 'induction chemotherapy'/exp OR 'chemotherapy, induction':ab,ti OR 'induction chemotherapy':ab,ti OR 'adjuvant chemotherapy'/exp OR 'chemotherapy, adjuvant':ab,ti OR 'adjuvant chemotherapy':ab,ti OR 'consolidation chemotherapy'/exp OR 'chemotherapeutic consolidation':ab,ti OR 'chemotherapy consolidation':ab,ti OR 'consolidation chemotherapy':ab,ti OR 'maintenance chemotherapy'/exp OR 'maintenance chemotherapy':ab,ti) | 1,446,730 |
| #3 | ('malignant neoplasm'/exp OR 'cancer':ab,ti OR 'cancers':ab,ti OR 'malignant neoplasia':ab,ti OR 'malignant neoplastic disease':ab,ti OR 'malignant tumor':ab,ti OR 'malignant tumour':ab,ti OR 'neoplasia, malignant':ab,ti OR 'neoplasmic malignancy':ab,ti OR 'neoplastic malignancy':ab,ti OR 'oncologic malignancy':ab,ti OR 'oncological malignancy':ab,ti OR 'tumor, malignant':ab,ti OR 'tumoral malignancy':ab,ti OR 'tumorous malignancy':ab,ti OR 'tumour, malignant':ab,ti OR 'malignant neoplasm':ab,ti OR 'oncology'/exp OR oncology:ab,ti OR 'neoplasm'/exp OR 'acral tumor':ab,ti OR 'acral tumour':ab,ti OR 'neoplasia':ab,ti OR 'neoplasms':ab,ti OR 'neoplastic disease':ab,ti OR 'neoplastic entity':ab,ti OR 'neoplastic mass':ab,ti OR 'tumor':ab,ti OR 'tumoral entity':ab,ti OR 'tumoral mass':ab,ti OR 'tumorous entity':ab,ti OR 'tumorous mass':ab,ti OR 'tumors':ab,ti OR 'tumour':ab,ti OR 'tumoural entity':ab,ti OR 'tumoural mass':ab,ti OR 'tumourous entity':ab,ti OR 'tumourous mass':ab,ti OR 'tumours':ab,ti OR 'neoplasm':ab,ti OR 'carcinoma'/exp OR carcinoma:ab,ti OR 'glioma'/exp OR glioma:ab,ti OR 'lymphoma'/exp OR lymphoma:ab,ti OR 'myeloma'/exp OR myeloma:ab,ti OR 'leukemia'/exp OR leukemia:ab,ti) | 7,318,701 |
| #4 | 'child'/exp OR 'adolescent'/exp OR 'children':ab,ti OR 'child':ab,ti OR 'pediatric':ab,ti OR 'teenager':ab,ti OR 'adolescent':ab,ti OR 'adolescence'/exp OR 'teenage':ab,ti OR 'adolescence':ab,ti OR 'minor (person)'/exp OR minor:ab,ti AND person:ab,ti OR 'juvenile'/exp OR 'youth':ab,ti OR 'juvenile':ab,ti OR 'young adult'/exp OR 'adult, young':ab,ti OR 'young adults':ab,ti OR 'young adult':ab,ti | 5,032,024 |
| #5 | evaluation*:ab,ti OR assessment*:ab,ti OR psychometric*:ab,ti OR measure*:ab,ti OR propert*:ab,ti OR develop*:ab,ti OR reliab*:ab,ti OR valid*:ab,ti OR responsive*:ab,ti OR method*:ab,ti OR tool*:ab,ti OR instrument*:ab,ti OR scale*:ab,ti OR survey*:ab,ti OR questionnaire*:ab,ti OR version*:ab,ti OR checklist*:ab,ti OR indicator*:ab,ti OR profile*:ab,ti OR index*:ab,ti OR indices*:ab,ti OR 'self report':ab,ti | 1,973,325 |
| #6 | #1 AND #2 AND #3 AND #4 AND #5 | 1,269 |

1. **PsycInfo (via ProQuest) (1806-, Search on 21, March, 2024)**

| **Concept** | **Search terms** | **n** |
| --- | --- | --- |
| #1 | main subject.Exact("peripheral neuropathy") OR tiab(peripheral neuropathy OR neurotoxicity chemotherapy OR chemotherapy induced peripheral neuropathy OR CIPN OR neurotoxicity OR neuropathy) | 12,101 |
| #2 | main subject.Exact("paclitaxel" OR "maintenance chemotherapy" OR "vincristine" OR "chemotherapy, adjuvant" OR "consolidation chemotherapy" OR "chemotherapy" OR "platinum" OR "vindesine" OR "vinblastine" OR "docetaxel" OR "carboplatin" OR "oxaliplatin" OR "etoposide" OR "cisplatin" OR "induction chemotherapy") OR tiab(chemotherapy OR chemotherapies OR vincristine OR vinblastine OR vinorelbine OR vindesine OR vincaalkaloids OR oxaliplatin OR eloxatin OR cisplatin OR carboplatin OR platinum OR taxane OR taxanes OR docetaxel OR paclitaxel OR etoposide OR vepesid OR gemcitabine OR induction chemotherapy OR chemotherapy adjuvant OR consolidation chemotherapy OR maintenance chemotherapy) | 8,088 |
| #3 | main subject.Exact("leukemia" OR "multiple myeloma" OR "carcinoma" OR "lymphoma" OR "glioma" OR "neoplasms" OR "oncology") OR tiab(cancer OR oncology OR tumor OR tumour OR carcinoma OR malignancy OR malignancies OR malignant OR neoplasms OR glioma OR lymphoma OR myeloma OR leukemia ) | 98,688 |
| #4 | main subject.Exact(“child” OR “pediatric” OR "minors" OR "adolescent" OR "young adult") OR tiab(child OR children OR pediatric OR adolescent OR adolescence OR teen OR teenager OR teens OR minor OR minors OR youth OR young adult) | 994,066 |
| #5 | tiab(evaluation* OR assessment* OR psychometric* OR measure* OR propert* OR develop* OR reliab* OR valid* OR responsive* OR method* OR tool* OR instrument* OR scale* OR survey* OR questionnaire* OR version* OR checklist* OR indicator* OR profile* OR index* OR indices* OR 'self report') | 3,126,523 |
| #6 | #1 AND #2 AND #3 AND #4 AND #5 | 25 |

1. **CINAHL (EBSCO) (1983-, Search on 21, March, 2024)**

| **Concept** | **Search terms** | **n** |
| --- | --- | --- |
| #1 | MH peripheral neuropathy in cancer patients OR MH neurotoxicity chemotherapy OR MH ( chemotherapy induced peripheral neuropathy or cipn or neurotoxicity or neuropathy or peripheral neuropathy ) OR TI peripheral neuropathy in cancer patients OR TI ( chemotherapy induced peripheral neuropathy or cipn or neurotoxicity or neuropathy or peripheral neuropathy ) OR TI neurotoxicity chemotherapy OR AB peripheral neuropathy in cancer patients OR AB neurotoxicity chemotherapy OR AB ( chemotherapy induced peripheral neuropathy or cipn or neurotoxicity or neuropathy or peripheral neuropathy ) | 24,246 |
| #2 | MH ( chemotherapy or chemo ) OR TI ( chemotherapy or chemo ) OR AB ( chemotherapy or chemo ) OR MH vincristine OR TI vincristine OR MH vinblastine OR TI vinblastine OR MH vinorelbine OR TI vinorelbine OR MH vinca alkaloids OR TI vinca alkaloids OR MH oxaliplatin neuropathy OR TI oxaliplatin neuropathy OR MH cisplatin OR TI cisplatin OR MH carboplatin OR TI carboplatin OR MH platinum OR TI platinum OR MH taxane OR TI taxane OR MH ( docetaxel or taxotere ) OR TI ( docetaxel or taxotere ) OR MH paclitaxel induced peripheral neuropathy OR TI paclitaxel induced peripheral neuropathy OR MH etoposide OR TI etoposide OR MH gemcitabine OR TI gemcitabine OR MH induction chemotherapy OR TI induction chemotherapy OR MH chemotherapy, adjuvant OR TI chemotherapy, adjuvant OR MH consolidation chemotherapy OR TI consolidation chemotherapy OR MH maintenance chemotherapy OR TI maintenance chemotherapy | 105,980 |
| #3 | MH ( glioma or brain tumor or glioblastoma ) OR TI ( glioma or brain tumor or glioblastoma ) OR MH ( lymphoma or leukemia ) OR TI ( lymphoma or leukemia ) OR MH carcinoma OR TI carcinoma OR MH ( neoplasms or neoplasm ) OR TI ( neoplasms or neoplasm ) OR MH ( neoplasms or oncology or cancer or tumor or malignancy ) OR TI ( neoplasms or oncology or cancer or tumor or malignancy ) OR MH ( myeloma or multiple myeloma or bone marrow cancer or myelomatosis ) OR TI ( myeloma or multiple myeloma or bone marrow cancer or myelomatosis ) | 619,445 |
| #4 | MH ( adolescents or child or teenagers or young adults or teen or youth or adolescence or minors ) OR TI ( child or pediatric or adolescents or teenagers or young adults or teen or youth or adolescence or minors ) OR MH adolescence OR TI adolescence | 1,105,459 |
| #5 | TI (evaluation* OR assessment* OR psychometric* OR measure* OR propert* OR develop* OR reliab* OR valid* OR responsive* OR method* OR tool* OR instrument* OR scale* OR survey* OR questionnaire* OR version* OR checklist* OR indicator* OR profile* OR index* OR indices* OR 'self report') | 851,020 |
| #6 | #1 AND #2 AND #3 AND #4 AND #5 | 22 |

1. **Scopus (1823-, Search on 21, March, 2024)**

| **Concept** | **Search terms** | **n** |
| --- | --- | --- |
| #1 | TITLE-ABS-KEY ( peripheral neuropathy OR neurotoxicity chemotherapy OR chemotherapy induced peripheral neuropathy OR CIPN OR neurotoxicity OR neuropathy ) | 5,697 |
| #2 | TITLE-ABS-KEY ( chemotherapy OR chemotherapies OR vincristine OR vinblastine OR vinorelbine OR vindesine OR vincaalkaloids OR oxaliplatin OR eloxatin OR cisplatin OR carboplatin OR platinum OR taxane OR taxanes OR docetaxel OR paclitaxel OR etoposide OR vepesid OR gemcitabine OR induction chemotherapy OR chemotherapy adjuvant OR consolidation chemotherapy OR maintenance chemotherapy) | 147,248 |
| #3 | TITLE-ABS-KEY ( cancer OR oncology OR tumor OR tumour OR carcinoma OR malignancy OR malignancies OR malignant OR neoplasms OR glioma OR lymphoma OR myeloma OR leukemia ) | 6,690,013 |
| #4 | TITLE-ABS-KEY (child OR children OR pediatric OR pediatrics OR adolescent OR adolescence OR teen OR teenager OR teens OR minor OR minors OR youth OR young adult) | 3,147,370 |
| #5 | TITLE-ABS- (evaluation* OR assessment* OR psychometric* OR measure* OR propert* OR develop* OR reliab* OR valid* OR responsive* OR method* OR tool* OR instrument* OR scale* OR survey* OR questionnaire* OR version* OR checklist* OR indicator* OR profile* OR index* OR indices* OR (self AND report)) | 55,037,765 |
| #6 | #1 AND #2 AND #3 AND #4 AND #5 | 51 |

1. **Web of science(1970-, Search on 21, March, 2024)**

| **Concept** | **Search terms** | **n** |
| --- | --- | --- |
| #1 | TS=(peripheral neuropathy OR neurotoxicity chemotherapy OR chemotherapy induced peripheral neuropathy OR cipn OR neurotoxicity OR neuropathy) | 177,726 |
| #2 | TS=(chemotherapy OR chemotherapies OR vincristine OR vinblastine OR vinorelbine OR vindesine OR vincaalkaloids OR oxaliplatin OR eloxatin OR cisplatin OR carboplatin OR platinum OR taxane OR taxanes OR docetaxel OR paclitaxel OR etoposide OR vepesid OR gemcitabine OR induction chemotherapy OR chemotherapy adjuvant OR consolidation chemotherapy OR maintenance chemotherapy) | 870,132 |
| #3 | TS=(cancer OR oncology OR tumor OR tumour OR carcinoma OR malignancy OR malignancies OR malignant OR neoplasms OR glioma OR lymphoma OR myeloma OR leukemia ) | 5,293,963 |
| #4 | TS=( child OR children OR pediatric OR adolescent OR adolescence OR teen OR teenager OR teens OR minor OR minors OR youth OR young adult) | 1,352,832 |
| #5 | TS=(evaluation* OR assessment* OR psychometric* OR measure* OR propert* OR develop* OR reliab* OR valid* OR responsive* OR method* OR tool* OR instrument* OR scale* OR survey* OR questionnaire* OR version* OR checklist* OR indicator* OR profile* OR index* OR indices* OR 'self report') | 35,667,533 |
| #6 | #1 AND #2 AND #3 AND #4 AND #5 | 714 |

1. **Cochrane Library (1996-, Search on 22, March, 2024)**

| **Concept** | **Search strategies** | **n** |
| --- | --- | --- |
| #1 | MeSH descriptor: [Peripheral Nervous System Diseases] explode all trees and with qualifier(s): [chemically induced - CI] OR ("chemotherapy induced peripheral neuropathy"):ti,ab,kw OR (peripheral nerve disease):ti,ab,kw OR (peripheral nerve disorder):ti,ab,kw AND (neurotoxicity):ti,ab,kw | 2754 |
| #2 | (chemotherapy):ti,ab,kw OR (vincristine):ti,ab,kw OR (vinorelbine):ti,ab,kw OR (cisplatin):ti,ab,kw OR (carboplatin):ti,ab,kw OR (platinum):ti,ab,kw OR (taxane):ti,ab,kw OR (docetaxel):ti,ab,kw OR (paclitaxel):ti,ab,kw OR (etoposide):ti,ab,kw OR (vepesid):ti,ab,kw OR (gemcitabine):ti,ab,kw OR (induction chemotherapy):ti,ab,kw OR (chemotherapy adjuvant):ti,ab,kw OR (consolidation chemotherapy):ti,ab,kw OR (maintenance chemotherapy):ti,ab,kw | 6428 |
| #3 | MeSH descriptor: [Neoplasms] explode all trees OR MeSH descriptor: [Carcinoma] explode all trees OR (Cancer*):ti,ab,kw OR (oncology*):ti,ab,kw OR (neoplasm*):ti,ab,kw OR (carcinoma*):ti,ab,kw OR (tumo*):ti,ab,kw OR (melanoma):ti,ab,kw OR (sarcoma):ti,ab,kw OR (adenocarcinoma*):ti,ab,kw OR (glioma*):ti,ab,kw OR (lymphoma*):ti,ab,kw OR (myeloma*):ti,ab,kw OR (leukemia*):ti,ab,kw OR (leucaemia*):ti,ab,kw | 294601 |
| #4 | MeSH descriptor: [Adolescent] explode all trees OR MeSH descriptor: [Child] explode all trees OR ("pediatric"):ti,ab,kw OR (child*):ti,ab,kw OR ("adolescence"):ti,ab,kw OR ("adolescent"):ti,ab,kw OR ("pediatric"):ti,ab,kw (Word variations have been searched) OR (teenager):ti,ab,kw OR (teens):ti,ab,kw OR (minor):ti,ab,kw OR (youth):ti,ab,kw OR (young adult):ti,ab,kw | 415296 |
| #5 | (evaluation):ti,ab,kw OR (assessment):ti,ab,kw OR (psychometric):ti,ab,kw OR (measure):ti,ab,kw OR (develop):ti,ab,kw OR (reliab*):ti,ab,kw OR (valid*):ti,ab,kw OR (responsive*):ti,ab,kw OR (instrument*):ti,ab,kw OR (tool*):ti,ab,kw OR (scale*):ti,ab,kw OR (survey*):ti,ab,kw OR (questionnaire*):ti,ab,kw OR (self report):ti,ab,kw | 1440933 |
| #6 | #1 and #2 and #3 AND #4 AND #5 | 11 |

**Appendix 2 Psychometric Property Definitions**

|  | **Psychometric concept and definition** | **Statistical test** | **Value** |
| --- | --- | --- | --- |
| **Reliability** |  |  |  |
| 1.Internal consistency | Internal consistency indicates how well the items on a tool fit together conceptually. | Cronbach’s alpha coefficient | ≥0.90 for clinical tools; ≥0.70 for research tools; Guideline based on underlying dimensions of the construct |
| 2.Test-retest reliability | Extent to which a tool measures stable characteristics at two separate times to the same group by administering the same test | Interclass Correlation Coefficients; Pearson Product Moment correlations; t test | High correlations; generally *r* ≥0 .70;  No statistically significant difference in scores from pre to posttest |
| 3.Alternative forms | Extent to which different forms of a tool yield comparable results when given to the same population during a single administration. | Pearson Product Moment Correlation Coefficient; Spearman Brown if test length has been changed (Both versions of the instrument must have equal means, variances, and alpha coefficients) | High correlations, generally r ≥0.70; |
| 4.Inter-rater (IRR) | Degree of agreement between two or more raters or observers when assessing subjects | Cohen’s Kappa (κ): categorical data;  Intraclass correlation coefficient: continuous data;  Percentage agreement | The Kappa statistic ranges from -1 to 1, where 1 indicates perfect agreement, 0 suggests no better agreement than chance, and -1 indicates complete disagreement. Positive rating: κ>0.40, negative rating: κ≤0.40  ICC values range between 0 and 1, with values closer to 1 indicating higher reliability. Excellent: 0.90-1.00, good: 0.75-0.90, moderate: 0.50-0.75, poor: <0.50.  / |
| 5.Intra-rater | Degree of agreement among repeated administrations of a diagnostic test performed by a single rater | Cohen’s Kappa: nominal data  Intraclass correlation coefficient: continuous data; |  |
| **Validity** |  |  |  |
| 1.Construct validity | The degree to which an instrument measures the construct it is intended to measure | Exploratory factor analysis (EFA);  Confirmatory factor analysis (CFA) | Eigenvalues >1.0;  Generally, factor loadings >0.40 |
| 2.Translational validity |  |  |  |
| Face | The instrument, on the face of it, appears to measure the construct | None | None: not considered a “true” measure of validity. Tool is accepted at face value |
| Content | Extent to which items in the tool sample the complete range of the attribute under study | Content validity ratio (CVR) or  Content validity index (CVI) | Depends on the number of expert reviewers |
| 3.Criterion validity | Evaluates how accurately a test measures the outcome it was designed to measure. | Item-total correlations, inter-item correlations | Substantial and high: *r* ≥0.45 is recommended by many authors |
| Concurrent | Scores on the tool are correlated to a related criterion at the same point in time. | High Pearson Product Moment correlations | Substantial and high: *r* ≥0.45 |
| Predictive | The degree to which test scores predict performance on some future criterion. | High Pearson Product Moment correlations | Substantial and high: *r* ≥0.45 |
| Convergent | Extent to which constructs that are theoretically similar correlate with one other. | High Pearson Product Moment correlations | Substantial and high: *r* ≥0.45 |
| Discriminant | Instrument’s capability to differentiate or discriminate between constructs that are theoretically different. | Low Pearson Product Moment correlations | Substantial and high: *r* ≤0.45 |
| **Sensitivity** | Extent to which the identification of differences among individual patients or patient groups at one point of time. | [True positive/ (True positive + false negative)]*100: ROC curves |  |
| **Responsiveness**  **(**longitudinal validity**)** | The ability of an instrument to accurately detect changes when it has occurred in individual patients or patient group. | Internal responsiveness: paired *t*-test, standardized effect size, standardized response mean (*SRM*), Guyatt’s responsiveness index.  External responsiveness: Area under the ROC curve, Correlation method (Pearson’s r and 95% confidence intervals), Linear regression method. | Negative rating: *SRM* < 0.20 |
| **Feasibility** | Easy to use. | Percentage of children completed the measure independently. |  |
